# The Impact of Lung Proteases on Snake-Derived Antimicrobial Peptides

**DOI:** 10.3390/biom11081106

**Published:** 2021-07-27

**Authors:** Shannice E. Creane, Simon R. Carlile, Damian Downey, Sinéad Weldon, John P. Dalton, Clifford C. Taggart

**Affiliations:** 1 Airway Innate Immunity Research (AiiR) Group, Wellcome-Wolfson Institute for Experimental Medicine, School of Medicine, Dentistry and Biomedical Sciences, Queen’s University Belfast, 97 Lisburn Road, Belfast BT9 7BL, UK; sfraser04@qub.ac.uk (S.E.C.); scarlile01@qub.ac.uk (S.R.C.); s.weldon@qub.ac.uk (S.W.); 2 Wellcome-Wolfson Institute for Experimental Medicine, School of Medicine, Dentistry and Biomedical Sciences, Queen’s University Belfast, 97 Lisburn Road, Belfast BT9 7BL, UK; d.downey@qub.ac.uk; 3 Zoology Department, School of Natural Sciences, Centre for One Health, Ryan Institute, National University of Ireland Galway, H91 TK33 Galway, Ireland; johnpius.dalton@nuigalway.ie; 4 School of Biological Sciences, Queen’s University Belfast, 97 Lisburn Road, Belfast BT9 7BL, UK

**Keywords:** antimicrobial peptide, protease, stability, antimicrobial, inflammation, anti-inflammatory

## Abstract

Respiratory infections are a leading cause of global morbidity and mortality and are of significant concern for individuals with chronic inflammatory lung diseases. There is an urgent need for novel antimicrobials. Antimicrobial peptides (AMPs) are naturally occurring innate immune response peptides with therapeutic potential. However, therapeutic development has been hindered by issues with stability and cytotoxicity. Availing of direct drug delivery to the affected site, for example the lung, can reduce unwanted systemic side effects and lower the required dose. As cystic fibrosis (CF) and chronic obstructive pulmonary disease (COPD) lungs typically exhibit elevated protease levels, the aim of this study was to assess their impact on snake-derived AMPs. Peptide cleavage was determined using SDS-PAGE and antimicrobial and anti-inflammatory activities of neutrophil elastase (NE)-incubated peptides were assessed using a radial diffusion assay (RDA) and an in vitro LPS-induced inflammation model, respectively. Although the snake-derived AMPs were found to be susceptible to cleavage by lung proteases including NE, several retained their function following NE-incubation. This facilitated the design of novel truncated derivatives that retained functionality following NE incubation. Snake-derived AMPs are tractable candidate treatments for use in environments that feature elevated NE levels, such as the CF airways.

## 1. Introduction

Respiratory infections are a leading cause of morbidity and mortality globally and are of significant concern for individuals with chronic inflammatory lung diseases, such as cystic fibrosis (CF) and chronic obstructive pulmonary disease (COPD), who are highly susceptible to lung infection [1]. Up to 95% of CF patients ultimately succumb to chronic lung infection [2]. Antimicrobial treatment is a mainstay component of the therapeutic strategy for CF. The emergence and increasing occurrence of antibiotic resistant bacteria necessitate the development of novel antimicrobial agents.

Antimicrobial peptides (AMPs) are naturally occurring peptides produced by plants and animals alike [3]. They are components of the innate immune response and possess potential as new antimicrobials due to their antimicrobial and/or immunomodulatory characteristics and broad spectrum of activity against bacteria, viruses, fungi and parasites [3]. Their immunomodulatory properties include pro- and anti-inflammatory effects, participation in chemotaxis, induction of cytokine and chemokine production, and dendritic cell maturation modulation [4,5]. Nevertheless, when considering a therapeutic for in vivo delivery, it is important to consider the local environment to which it will be exposed. Availing of direct drug delivery to the affected site can reduce unwanted systemic side effects and reduce the required dose, which is attractive for expensive drugs such as synthetic AMPs [6]. Unfortunately, the therapeutic development of AMPs has been hindered by issues with stability and cytotoxicity in vivo. 

Airway mucus accumulation is evident in chronic lung diseases such as asthma, COPD and CF [7]. A change in the balance of water-secreting and mucus-secreting elements leads to excessive viscous mucous secretions that are more challenging for the cilia to mobilise, resulting in mucus accumulation [7,8]. Neutrophil migration through highly viscous mucous is more difficult, leading to their entrapment and the uncontrolled release of proteases, reactive oxygen species, and other pro-inflammatory components into the microenvironment, leading to further immune cell recruitment [9]. As a result, protease levels, such as neutrophil elastase (NE), are higher in CF patients than normal subjects [10,11]. Endogenous AMPs, such as LL-37, have been found to be readily degraded by proteases including NE, and cathepsins B and K, leading to impaired antimicrobial activity [11,12,13]. Thus, elevated protease levels contribute to the impaired ability of AMPs to combat infection in the CF lung. Development of an exogenous AMP that is either resistant to proteolytic degradation or that is functional in the presence of proteases is of therapeutic interest in the localised treatment of infection of the chronically inflamed lungs. AMPs originally derived from *Hydrophis cyanocinctus* (Sea snake) (known in this study as Sn1), *Ophiophagus hannah* (King Cobra) (known in this study as SnE1) and *Crotalus durissus terrificus* (South America Rattlesnake) (known in this study as SnV1) were of particular interest in this study. These peptides have previously been shown to exhibit potent antimicrobial activity against a wide range of bacterial strains, including clinical isolates [14,15,16,17]. Furthermore, Sn1, SnV1 and a truncated derivative of SnE1 have previously been shown to modulate the inflammatory response in the presence of pro-inflammatory stimuli [14,16,18]. While Sn1 has been shown to inhibit the LPS-induced inflammatory response via direct LPS binding and via binding to the myeloid differentiation protein-2 (MD2) of the Toll-like receptor 4 (TLR4)-MD2 complex, an exact mechanism has not been established for SnE1 or SnV1 [14]. Furthermore, our group previously reported that Sn1 held therapeutic potential for infection due to antimicrobial and anti-inflammatory effects observed in vivo [19]. Thus, assessing the therapeutic potential of these snake-derived peptides in the context of inflammatory lung disease was of interest due to their antimicrobial and/or immunomodulatory properties. In this study, the impact of lung proteases on snake-derived AMPs was assessed and we show that despite these AMPs being susceptible to protease degradation, several fragments still retained their antimicrobial activity which allowed the design of smaller stable derivative peptides.

## 2. Materials and Methods

### 2.1. Peptide Synthesis

Peptides were manufactured by Fmoc synthesis in free acid form upon request by GL biochem (Shanghai, China). Batches of peptide were at least 95% purity and validated by high performance liquid chromatography (HPLC) and mass spectrometry. Peptides were aliquoted and lyophilised into 1 mg vials prior to delivery and stored at −20 °C upon receipt. 

### 2.2. Peptide Predicted Physiochemical Properties, Secondary Structures and Cleavage Sites

The physiochemical properties of the peptides were predicted using ExPASy Bioinformatics Resource Portal Protparam software (Swiss Institute of Bioinformatics, Lausanne, Switzerland) available at https://web.expasy.org/protparam/ (accessed on 26 July 2021). Helical wheel projections were produced using Heliquest software (version 1.2, Institute Pharmacology Moléculaire Et Cellulaire, Valbonne, France) available at http://heliquest.ipmc.cnrs.fr/cgi-bin/ComputParams.py (accessed on 26 July 2021). Secondary structure and corresponding 3D model predictions were generated using I-TASSER software (University of Michigan, Ann Arbor, MI, USA) available at https://zhanglab.ccmb.med.umich.edu/I-TASSER/ (accessed on 26 July 2021 (Roy et al., 2010; Yang Zhang, 2008)). Peptide cleavage sites were determined by ExPASy PeptideCutter (Swiss Institute of Bioinformatics, Lausanne, Switzerland) available at https://web.expasy.org/peptide_cutter/ (accessed on 26 July 2021) and Prosper software (Monash University, Melbourne, Australia) available at https://prosper.erc.monash.edu.au/ (accessed on 6 April 2020) [20].

### 2.3. CF Sputum

Sputum from Pseudomonas-infected CF patients was obtained anonymously from the adult CF Centre at Belfast City Hospital. Sputum samples were in excess to requirements for diagnostic purposes. Permission to use sputum samples, which would have been disposed of, for validation purposes was given by the Director of R&D, Belfast Health and Social Care Trust. Sputum was frozen immediately at −80 °C and used the next day after thawing at room temperature.

### 2.4. Peptides-CF Sputum Incubations

An aliquot of 500 μg/mL of snake AMPs Sn1, Sn1a, Sn1b, SnE1 or SnV1, or the parental molar equivalent of Sn1bN, SnE1N or SnE1-F was incubated with or without CF sputum (diluted 100-fold in 1X tris-buffered saline (TBS)) for four hours. SnE1, SnE1N and SnE1-F were also incubated with CF sputum for 24 h due to superior stability compared to the other peptides. Samples were transferred to a fresh Eppendorf at regular time-points (0, 1, 2, 3, 4, 18 and 24, as appropriate) and boiled on a heat block at 95 °C for ten minutes to terminate the reaction. Incubations were also carried out in the presence of cysteine, MMP and serine protease inhibitors (E64 (Thermo Fisher Scientific, Loughborough, UK), EDTA (Thermo Fisher Scientific, Loughborough, UK) and pefabloc (Merck, Gillingham, UK), respectively) to determine the class of proteases responsible for observed peptide degradation. CF sputum (diluted 100-fold in 1X TBS) was incubated with 0.1 mM E64, 5 mM EDTA or 1 mM pefabloc for 30 min prior to addition of peptides. Following the addition of peptide, the samples were incubated for four or 24 h at 37 °C. An aliquot was taken at 0, 4 or 24 h and boiled for ten minutes at 95 °C to terminate the reaction. Samples were stored at 4 °C until analysed by SDS-PAGE.

### 2.5. Peptide-Neutrophil Elastase Incubations

An aliquot of 500 μg/mL of Sn1, Sn1a, Sn1b, SnE1 or SnV1, or the parental molar equivalent of Sn1bN, SnE1N or SnE1-F was incubated with 500 nM human sputum leucocyte elastase (Elastin Products Company, Inc., Owensville, MO, USA) at 37 °C for four hours with samples taken at hourly time-points from zero to four hours. The method for reaction termination was dependent on subsequent experiments/analysis. For samples intended for investigation of peptide activity using in vitro experiments the reaction was terminated via addition of 1 mM of the serine protease inhibitor pefabloc (Merck, Gillingham, UK), which was added to all groups including the NE alone groups. Samples intended for peptide degradation analysis via SDS-PAGE or mass spectrometry were boiled at 95 °C for ten minutes using a heat block to terminate the reaction.

### 2.6. Analysis by SDS-PAGE

Peptide samples were separated by sodium dodecyl sulphate-polyacrylamide gel electrophoresis (SDS-PAGE) under denaturing and reducing conditions using 4–12% Invitrogen NuPAGE Novex Bis-Tris precast gels (Thermo Fisher Scientific, Loughborough, UK) and the Invitrogen XCell SureLock Mini-Cell (Thermo Fisher Scientific, Loughborough, UK). An aliquot of 10 μL peptide samples was incubated with 4 μL NuPAGE lithium dodecyl sulphate (LDS) sample buffer (Thermo Fisher Scientific) and 2 μL of 500 nM dithiothreitol (DTT) (Fluorochem, Hadfield, UK) for ten minutes at 95 °C and 15 μL of sample mixture loaded per lane and 5 μL of SeeBlue Plus2 pre-stained protein standard (Thermo Fisher Scientific, Loughborough, UK) loaded in at least one lane. The samples were run using 1X 2-morpholinoethanesulfonic acid (MES)-SDS running buffer (Thermo Fisher Scientific, Loughborough, UK). Electrophoresis was performed under a constant current of 125 mA for 40–60 min. The gel was stained using Biosafe Coomassie Brilliant Blue G-250 stain (Biorad, Watford, UK) for 60 min, washed and destained in water overnight and imaged using a Syngene G:box and GeneSnap software (Syngene, Cambridge, UK).

### 2.7. Mass Spectrometry

Aliquots of 500 μg/mL of Sn1b, SnE1 and SnV1 incubated with/without 500 nM of NE in 1 X TBS for four hours at 37 °C were analysed by Matrix-Assisted Laser Desorption/Ionisation-Time of Flight Mass Spectrometry (MALDI-TOF-MS). This service was provided by the University of Dundee (Dundee, UK).

### 2.8. Radial Diffusion Assay

A radial diffusion assay (RDA) was employed to assess the antimicrobial activity of the peptides using a previously described method [21]. Briefly, a 100 μL aliquot of an overnight culture of *P. aeruginosa* 27853 was diluted in 25 mL of Mueller Hinton Broth and incubated aerobically at 37 °C in a ZHWY-11C shaking incubator for three hours to obtain a mid-logarithmic culture. The culture was centrifuged at 2594× *g* for 10 min, washed twice with 5 mL of 10 mM sodium phosphate buffer and the optical density at a wavelength of 600 nm (OD600 nm) adjusted to 0.4–0.5 in sterile 10 mM sodium phosphate buffer. An aliquot of 100 μL of this bacterial suspension (equating to approximately 5 × 10^6^ bacterial cells) was added to 10 mL of molten base agarose (21 mg MHB powder (Biokar Diagnostics, Pantin, France), 1 g agarose (Merck, Gillingham, UK), 20 μL Tween 20 (Merck, Gillingham, UK), 100 mL sodium phosphate buffer (10 mM), inverted, poured onto a square Petri dish (Sarstedt, Nümbrecht, Germany) and allowed to solidify. Wells of diameter 2.3 mm were punched into the agarose using a suction pump. A two-fold serial dilution (concentrations indicated in figures) of peptide was added to the plate, with 3 μL of each test concentration added per well. The insect AMP, cecropin A (100 μg/mL) (Merck, Gillingham, UK), and vehicle controls, were included as positive and negative controls, respectively. The Petri dish was incubated upright at 37 °C for three hours to allow diffusion of test compounds into the agarose. Then, 10 mL of molten high nutrient overlay agarose (4.2 g MHB powder, 1 g agarose, 100 mL distilled water) was poured over the base agarose, allowed to solidify and incubated aerobically at 37 °C overnight. The following day, 5 mL of conditioning medium (10 mL acetic acid (Merck, Gillingham, UK), 2 mL DMSO, (Merck, Gillingham, UK), 88 mL distilled water) was added to the Petri dish for 10 min with gentle rotation to prevent any further bacterial growth. The conditioning medium was replaced with Coomassie brilliant blue stain (2 mg Coomassie Brilliant Blue R250 (Merck, Gillingham, UK), 27 mL methanol, (Merck, Gillingham, UK), 15 mL 37% formaldehyde (Merck, Gillingham, UK), 63 mL distilled water) and incubated overnight at room temperature with gentle rotation. Inhibition of bacterial growth was indicated by circular areas devoid of colour. Measurement of the diameter of these areas was performed using a x8 measuring eyepiece (Flubacher, Wingate, UK). The Petri dish was imaged using a Syngene G:box and GeneSnap software. Minimal inhibitory concentration (MIC) values were determined by linear regression of the size of inhibition zones versus the log concentration of the peptides. 

### 2.9. LPS Stimulation with THP-1 Monocyte-Derived Macrophages

THP-1 cells (human acute monocytic leukaemia cells) were purchased from the European Collection of Authentic Cell Culture (ECACC) and maintained at cell density of 2–9 × 10^5^ cells/mL in Roswell Park Memorial Institute (RPMI) 1640 medium (Thermo Fisher Scientific, Loughborough, UK) supplemented with 10% heat-inactivated foetal bovine serum (FBS) (Thermo Fisher Scientific, Loughborough, UK) and 2 mM L-glutamine (Thermo Fisher Scientific, Loughborough, UK) in a humidified incubator with 5% CO_2_ at 37 °C [22]. A previously described method of LPS-induced inflammation using THP-1 monocyte-derived macrophages was employed to assess the anti-inflammatory activity of the peptides [19]. THP-1 cells were seeded at a density of 2.5 × 10^5^ cells/well in a 24-well plate (Thermo Fisher Scientific, Loughborough, UK) and differentiated to THP-1 monocyte-derived macrophages by culturing under standard cell culture conditions (37 °C/5% CO_2_) in the presence of 160 nM phorbol 12-myristate 13-acetate (PMA) (Merck, Gillingham, UK) for 72 h. The cell supernatant was replaced with 1 mL of fresh media and incubated for a further 24 h prior to experimentation. Immediately prior to cell stimulation, cell supernatants were replaced with 500 μL of fresh RPMI (+10% FBS, 2 mM L-glutamine). The cells were incubated with peptide (concentrations indicated in figures) and/or 100 ng/mL Pseudomonas aeruginosa LPS (Serotype 10, Source strain ATCC 27316) (Merck, Gillingham, UK) for 16 h under standard cell culture conditions. The cell supernatants were collected for cytokine quantification.

### 2.10. Enzyme Linked Immunosorbent Assay (ELISA)

The IL-6 and IL-8 concentrations in the cell supernatants were quantified using the IL-6 and IL-8 ELISAs (Thermo Fisher Scientific, Loughborough, UK), and were performed as per the manufacturers’ instructions.

## 3. Results

### 3.1. Predicted Physiochemical Properties of Peptides

The molecular weight (Da), net charge, hydrophobic moment and Grand Average of Hydropathicity (GRAVY) value of the peptides used in this study were predicted using ExPASy Bioinformatics Resource Portal Protparam software (Swiss Institute of Bioinformatics, Lausanne, Switzerland) available at https://web.expasy.org/protparam/ (accessed on 26 July 2021) (Table 1). All peptides of interest incorporate a large proportion of positively charged amino acids such as lysine and arginine, resulting in a highly positive net charge. All peptides were predicted to exhibit an α-helical secondary structure, and to be highly amphipathic, with polar and non-polar amino acids forming hydrophobic and hydrophilic faces on opposing sides.

### 3.2. Peptides Are Susceptible to Degradation by Sputum Enzymes

**Figure 1:**
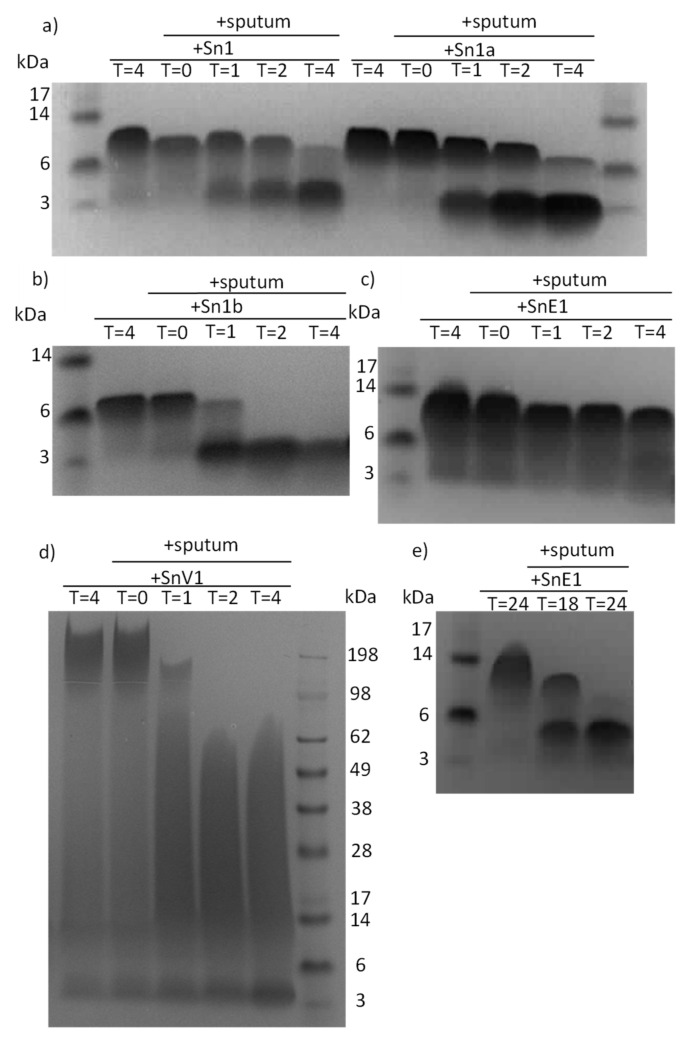
SDS-PAGE gels of peptides incubated with CF sputum. (**a**) Sn1, Sn1a, (**b**) Sn1b, (**c**) SnE1 and (**d**) SnV1 were incubated with CF sputum at 37 °C for 0–4 h, with samples taken at hourly time points. (**e**) SnE1 was incubated with CF sputum at 37 °C for 18–24 h, with samples taken at 18 and 24 h. Samples then underwent electrophoresis and stained with Coomassie stain to allow visualisation.

To determine if peptides were susceptible to degradation in the presence of lung proteases, peptides were incubated with CF sputum. Upon electrophoresis, the peptides were found to migrate with an apparent molecular weight that was larger than their theoretical molecular weight. This may be due to the presence of polar residues [23]. It has been demonstrated previously that polar amino acid residues in transmembrane helices can affect the interaction between peptide and SDS, resulting in reduced gel migration and a higher apparent molecular weight than the theoretical molecular weight [23]. Electrophoresis and Coomassie staining revealed that Sn1, Sn1a, Sn1b and SnV1 were highly susceptible to degradation in the presence of either sputum within 4 h (Figure 1a,b,d). SnE1 appeared to be more resistant to degradation in the first 4 h, but underwent significant degradation within 18 h and almost complete degradation by 24 h (Figure 1c,e).

**Figure 2:**
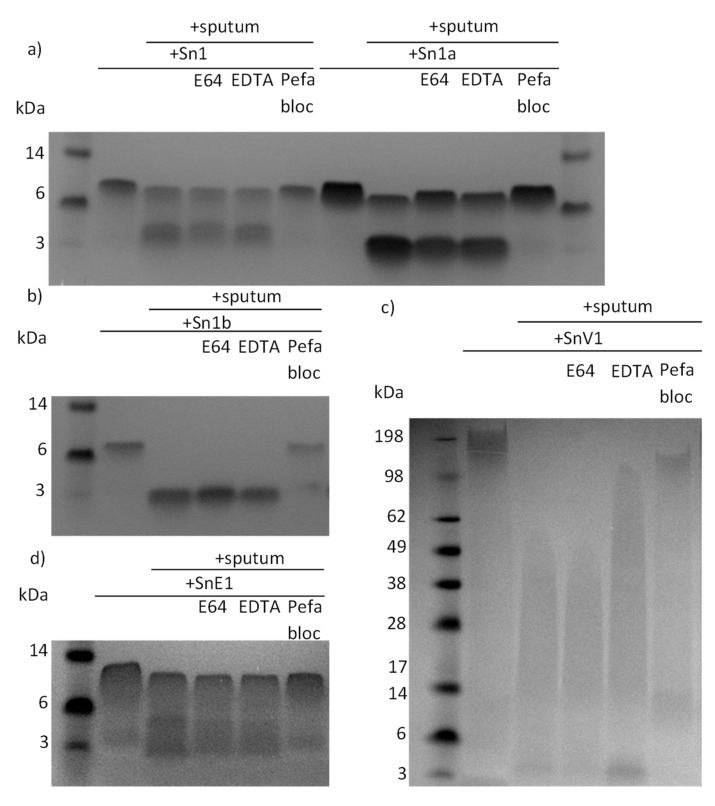

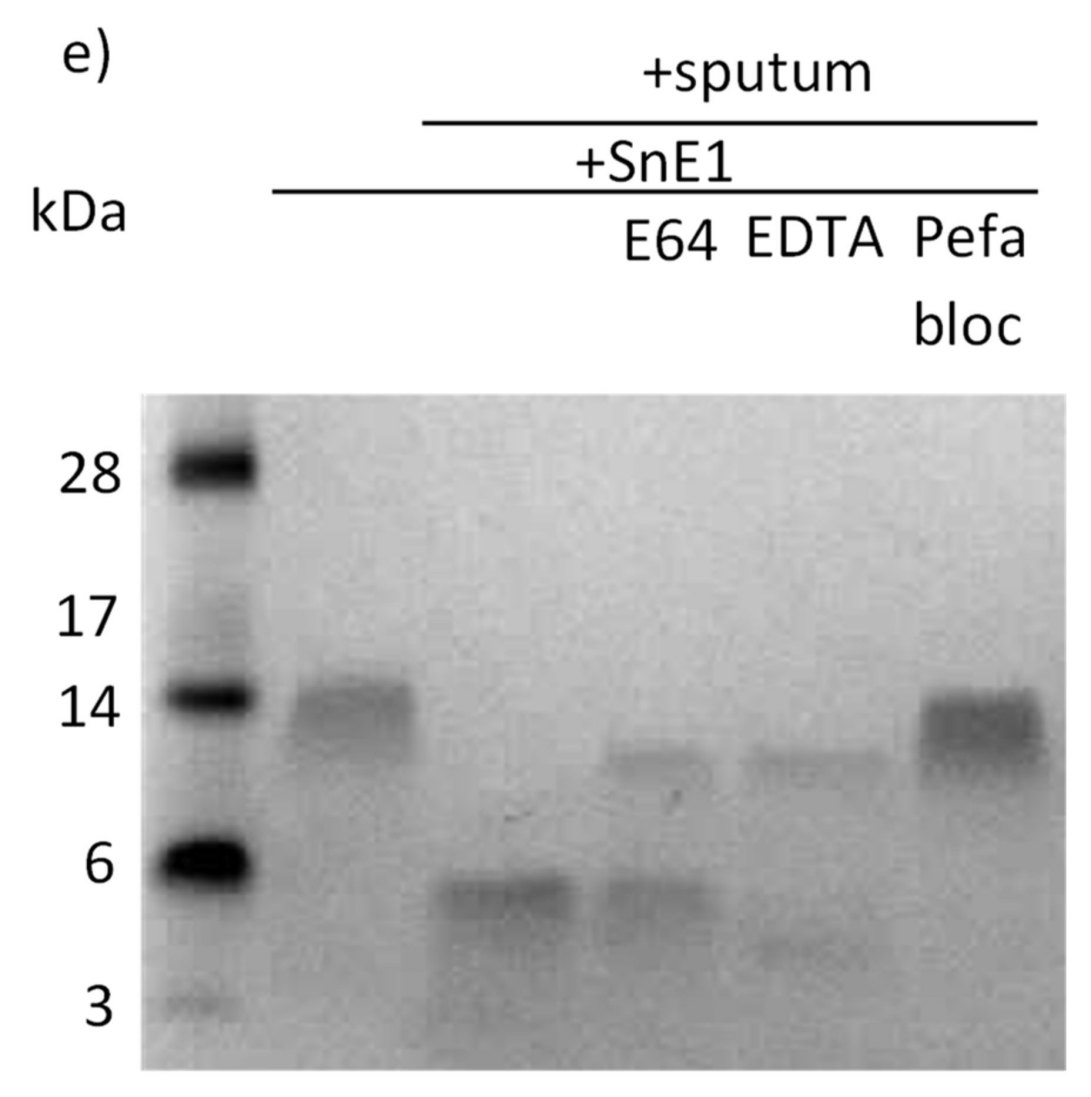
SDS-PAGE gels of peptides incubated with CF sputum in the presence/absence of protease inhibitors. An aliquot of 500 μg/mL of (**a**) Sn1, Sn1a, (**b**) Sn1b (**c**) SnV1, or (**d**) SnE1 was incubated with CF sputum in the presence/absence of E64 (cysteine protease inhibitor), EDTA (MMP inhibitor) or pefabloc (serine protease inhibitor) for 4 h. An aliquot of 500 μg/mL of (**e**) SnE1 was also incubated with CF sputum and inhibitors for 24 h. The samples underwent electrophoresis and stained with Coomassie stain to allow visualisation.

To investigate protease families involved in peptide degradation, we ran a series of peptide incubations with sputum samples from patients with CF in the presence/absence of MMP, serine or cysteine protease inhibitors. Sn1, Sn1a, Sn1b and SnV1 exhibited considerable degradation within four hours of incubation with CF sputum and this effect was prevented only by the inhibitor pefabloc, suggesting that a serine protease was responsible for the degradation of the peptides (Figure 2a,b,d). SnE1 exhibited resistance to degradation by sputum proteases compared to the other test peptides, with little degradation after four hours but even this peptide was completely degraded after 24 h (Figure 2c). Incubation with pefabloc prevented this effect, suggesting that serine proteases were responsible for degradation. E64 or EDTA also appeared to offer some protection from degradation by cysteine proteases and MMPs, respectively (Figure 2e), implicating cysteine proteases and MMPs in the degradation of SnE1 as well.

### 3.3. Peptides Are Susceptible to Degradation by Neutrophil Elastase

**Figure 3:**
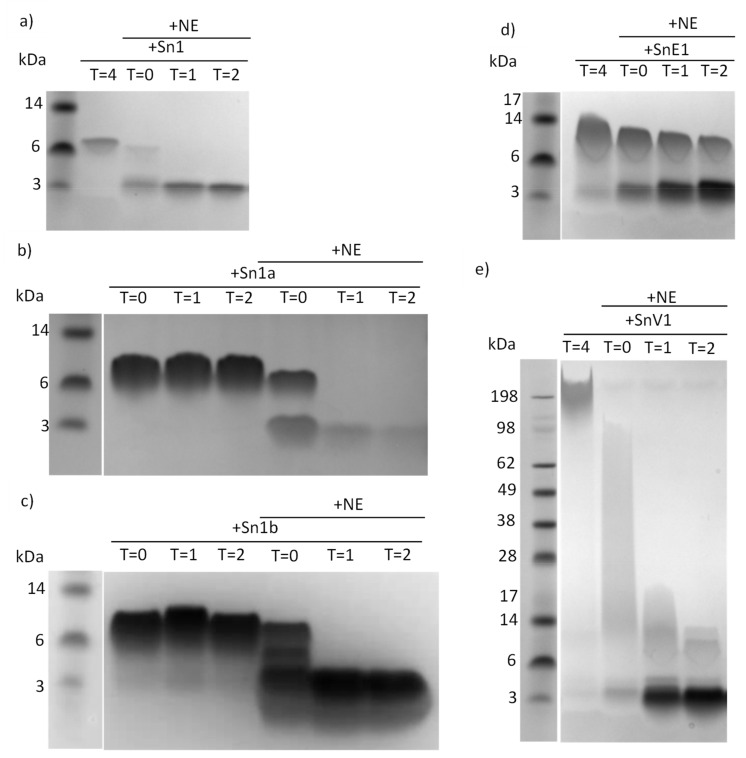
SDS-PAGE gels of peptides incubated with NE. An aliquot of 500 μg/mL of (**a**) Sn1, (**b**) Sn1a, (**c**) Sn1b, (**d**) SnE1 or (**e**) SnV1 was incubated with 500 nM of neutrophil elastase for 0–4 h. Samples were electrophoresed and stained with Coomassie stain.

As sputum incubations suggested that serine proteases were most likely responsible for peptide degradation and cleavage, we next ran a series of peptide incubations with the serine protease neutrophil elastase. Analysis by electrophoresis and Coomassie staining demonstrated that all peptides tested were cleaved rapidly following addition of NE (Figure 3).

### 3.4. The Effect of NE Incubation on Antimicrobial Activity of Peptides

To determine if cleavage by NE altered antimicrobial activity of the peptides, peptides were tested against *P. aeruginosa* 27853 following incubation of the peptides with NE for four hours. All peptides tested exhibited an increase in the mean MIC value (Table 2). NE-incubated Sn1a exhibited the most dramatic reduction in antimicrobial activity against *P. aeruginosa* 27853, exhibiting an increase in mean MIC value from 0.660 μM to 45.6 μM. Of the Sn1-derived peptides, Sn1b retained the most antimicrobial activity, with the smallest change in mean MIC value. However, SnE1 demonstrated the least change in mean MIC values against *P. aeruginosa* 27853 following incubation with NE, with an increase from 3.25 μM to 5.07 μM. While all peptides exhibited increased mean MIC values, only SnV1 yielded statistical significance when challenged with *P. aeruginosa* 27853.

### 3.5. The Effect of NE Incubation on Peptide Anti-Inflammatory Activity

**Figure 4:**
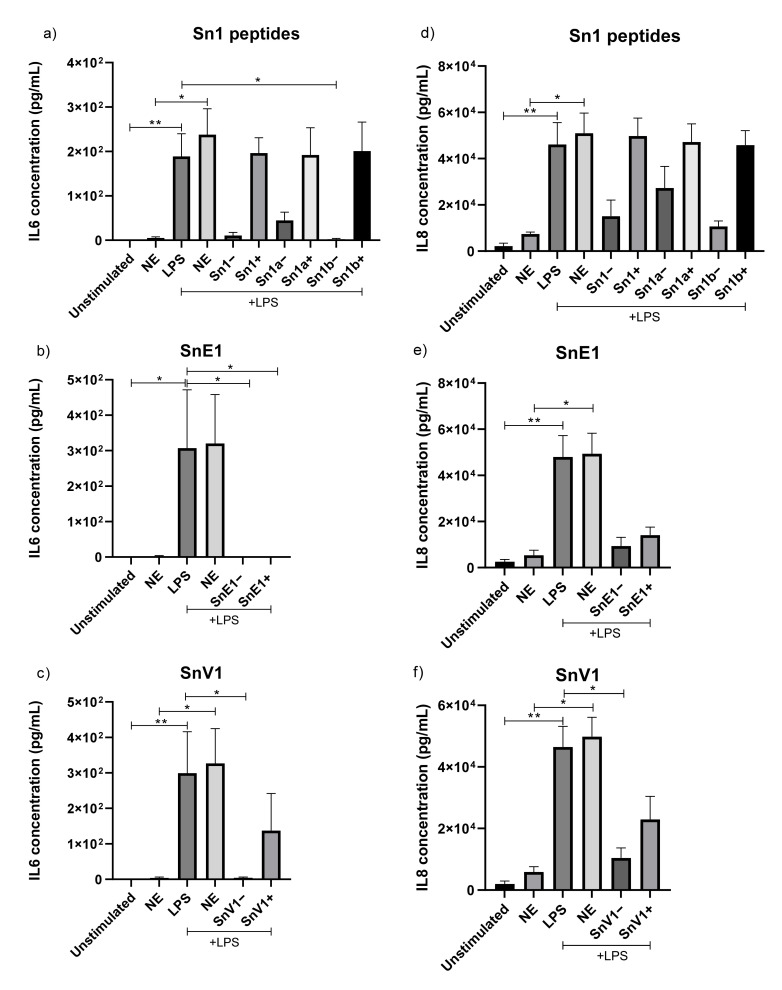
IL-6 and IL-8 concentrations in the supernatant of THP-1 monocyte-derived macrophages. THP-1 monocyte-derived macrophages were incubated with 5 μg/mL of NE-incubated or control (**a**,**d**) Sn1, Sn1a, Sn1b, (**b**,**e**) SnE1, (**c**,**f**) SnV1 and/or 100 ng/mL of LPS for 16 h. (**a**–**c**) IL-6 and (**d**–**f**) IL-8 levels in cell supernatants were then measured via ELISA. Kruskal–Wallis with Dunn’s Multiple Comparison * *p* < 0.05, ** *p* < 0.01, *n* = 3–4. +/− = incubation with/without NE immediately prior to the experiment.

To determine if the NE incubation influenced anti-inflammatory activity of the peptides, NE-incubated peptides were tested in an in vitro LPS-induced inflammation model using THP-1 monocyte-derived macrophages. Sn1b, SnE1 and SnV1 incubated in the absence of NE prior to cell stimulation resulted in significant reduction in LPS-induced IL-6, (Figure 4a–c). Incubation of NE-incubated SnE1 with THP-1 cells resulted in significant reduction in LPS-induced IL-6 concentration, whereas NE-incubated Sn1, Sn1a and Sn1b did not alter LPS-induced IL-6 levels.

Only SnV1 incubated in the absence of NE prior to cell stimulation resulted in a statistically significant decrease in LPS-induced IL-8 level (Figure 4f). The other NE-incubated peptides had no statistically significant effect on LPS-induced IL-8 release by THP-1 cells, but NE-incubated and non-NE-incubated SnE1 generated trends toward reduced IL-8 (Figure 4d,e). 

### 3.6. Identification of Active Portions of Peptides Using Mass Spectrometry

**Figure 5:**
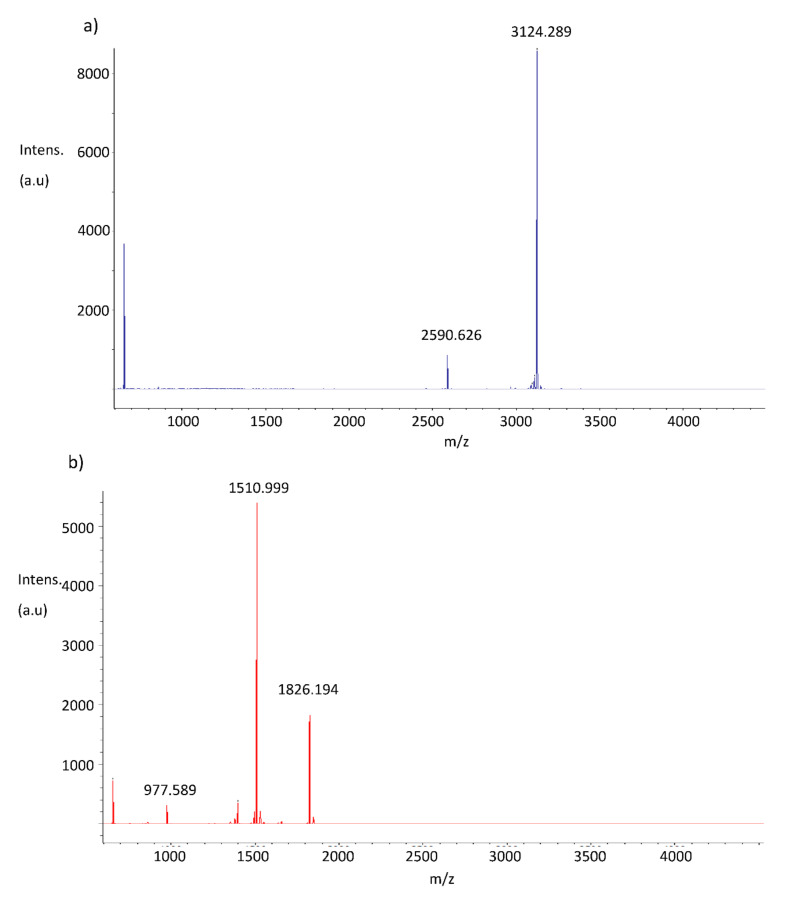

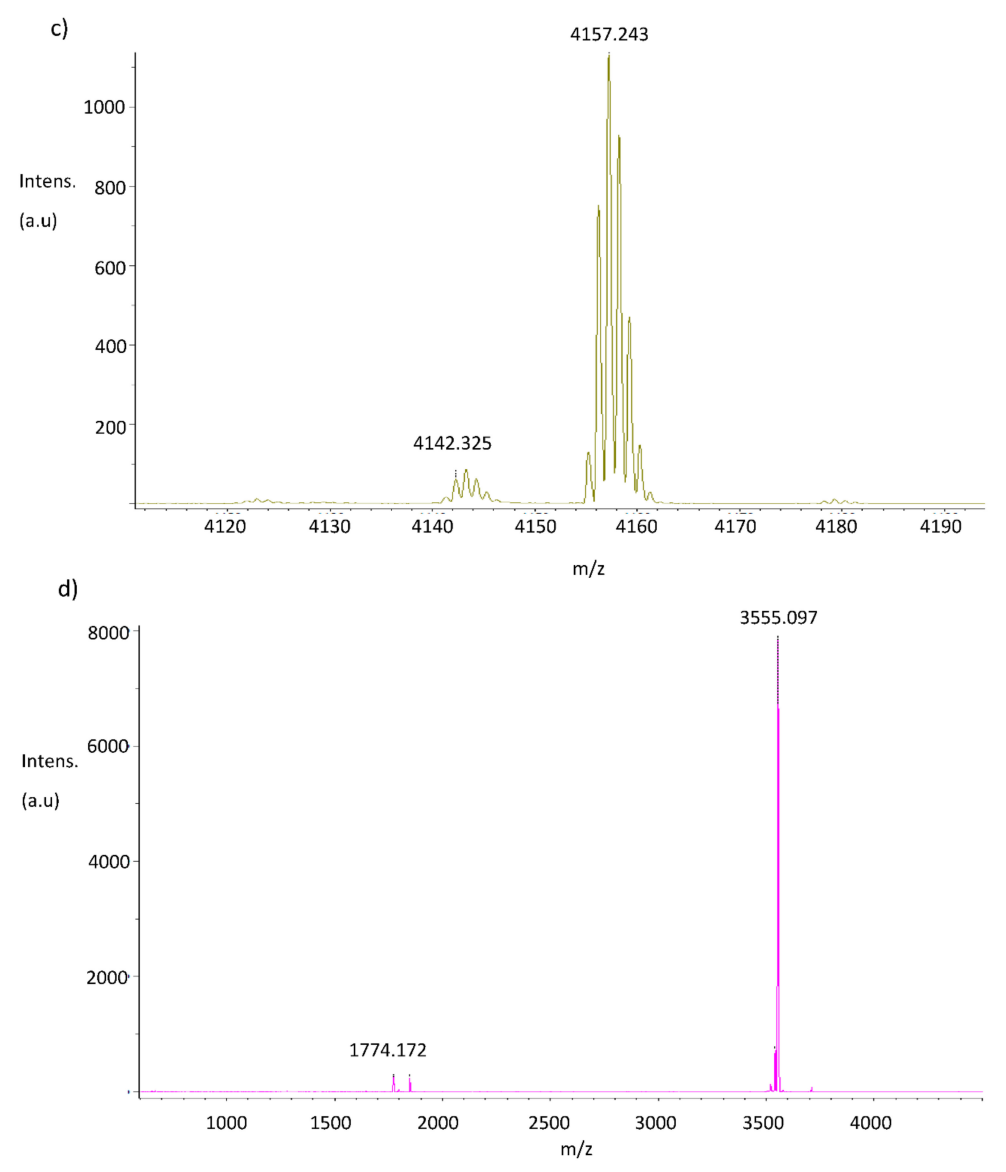

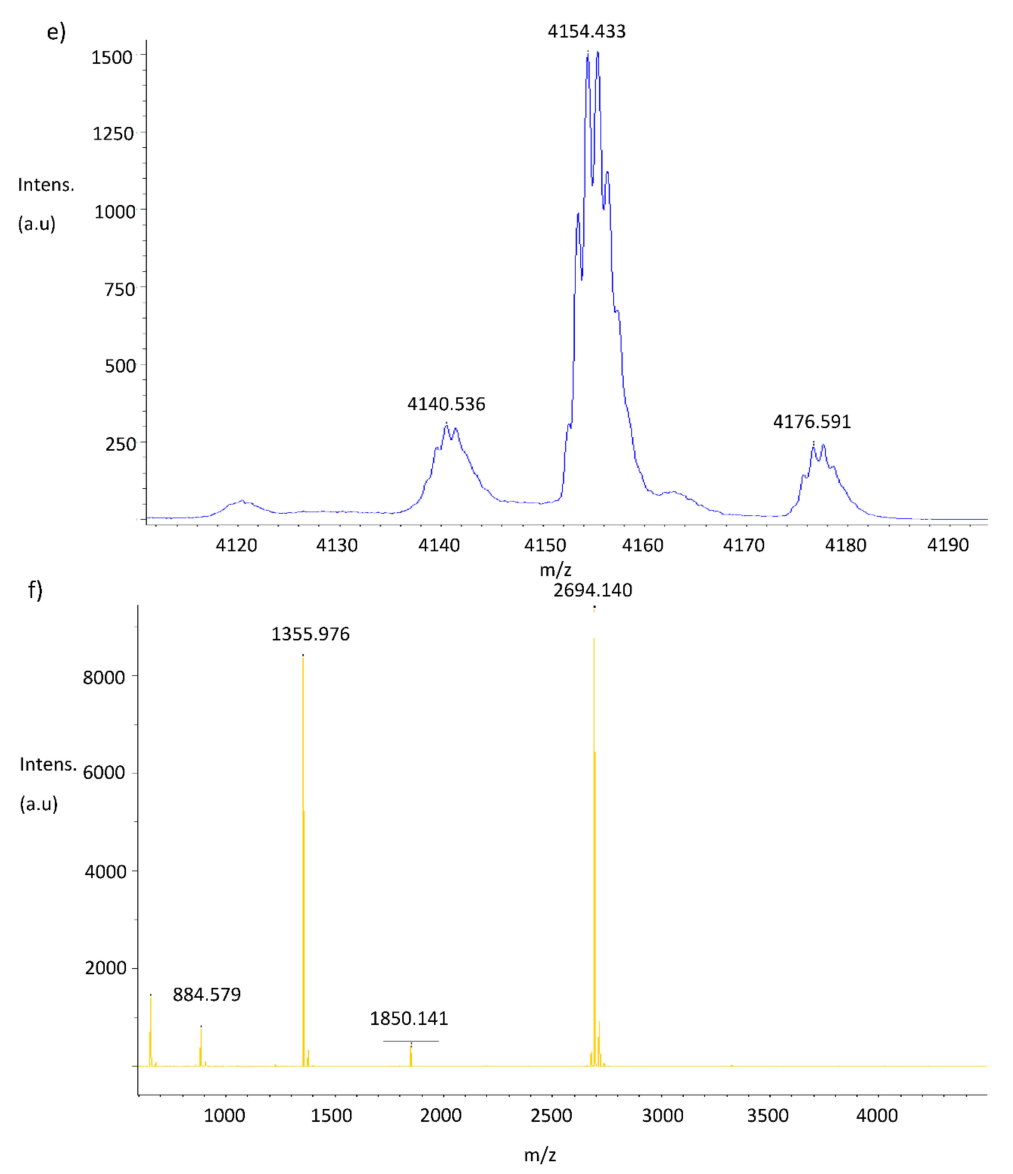
Mass spectrometry profiles of NE-incubated peptides. Mass spectrometry profiles of (**a**,**b**) Sn1b, (**c**,**d**) SnE1 and SnV1 (**e**,**f**) following incubation in the presence (**b**,**d**,**f**) or absence (**a**,**c**,**e**) of NE for 4 h. The relative intensity of the ions (arbitrary units, au) is shown on the y-axis, and the mass to charge ratio (*m*/*z*), corresponding to the molecular masses (Daltons) are shown on the x-axis.

Having determined that Sn1b, SnE1 and SnV1 retained antimicrobial and/or anti-inflammatory function following NE incubation, we next determined the peptide segments responsible for these activities using mass spectrometry (Figure 5). Comparison of NE-incubated and parent peptide mass spectra profiles facilitated identification of NE cleavage sites (Figure 5 and Table 3).

### 3.7. Peptides Derivatives and Stability in CF Sputum

Guided by in vitro cleavage findings shown in Table 3, truncated Sn1b and SnE1 derivatives were developed with the aim of improving peptide stability in the presence of NE, whilst maintaining peptide functionality (Table 1). As SnE1 exhibited better retained anti-inflammatory activity than SnV1 following cleavage by NE and as SnV1 cleavage resulted in several small fragments, SnV1 derivatives were not generated. 

**Figure 6:**
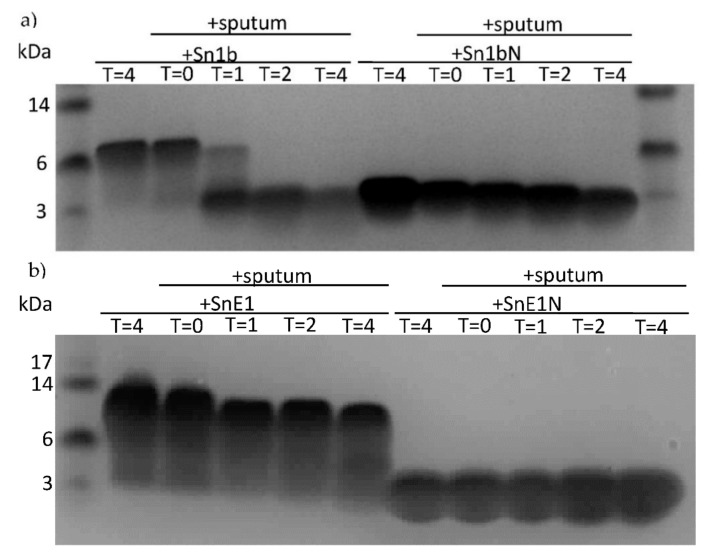

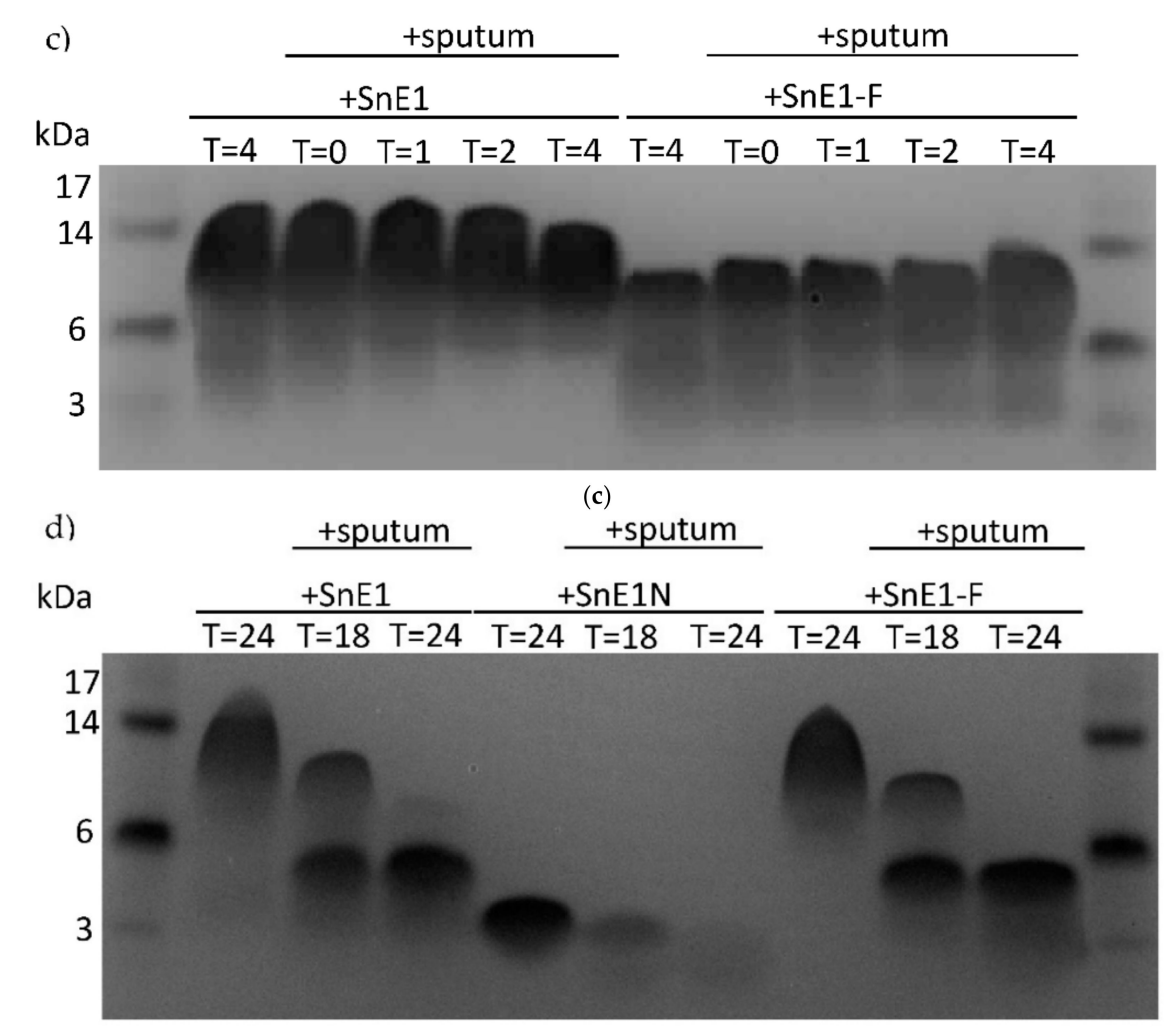
SDS-PAGE gels of next generation derivative peptides following incubation with CF sputum. An aliquot of (**a**) Sn1b, Sn1bN (160 μM), (**b**) SnE1, SnE1N (120 μM), (**c**) SnE1 and SnE1-F (120 μM) were incubated with CF sputum for 0–4 h. (**d**) SnE1, SnE1N and SnE1-F (120 μM) were incubated with CF sputum for 0–24 h. Samples were electrophoresed and stained with Coomassie stain for visual analysis.

When directly compared to respective parent peptides in CF sputum incubations, two derivatives were found to be susceptible to degradation by sputum proteases (Figure 6). Incubation of Sn1bN with CF sputum did not result in obvious degradation under the test conditions, whereas cleavage of Sn1b was clearly evident, suggesting Sn1bN may be more stable in the presence of lung proteases compared to parent Sn1b (Figure 6a). As the degradation of SnE1 and derivatives was not obvious following incubation with sputum for 4 h, the incubation time of these peptides was increased to 24 h (Figure 6b,c). SnE1, SnE1N and SnE1-F exhibited transition from one band of higher molecular weight to one of lower molecular weight after incubation with sputum for 24 h indicative of degradation by proteases (Figure 6d). 

**Figure 7:**
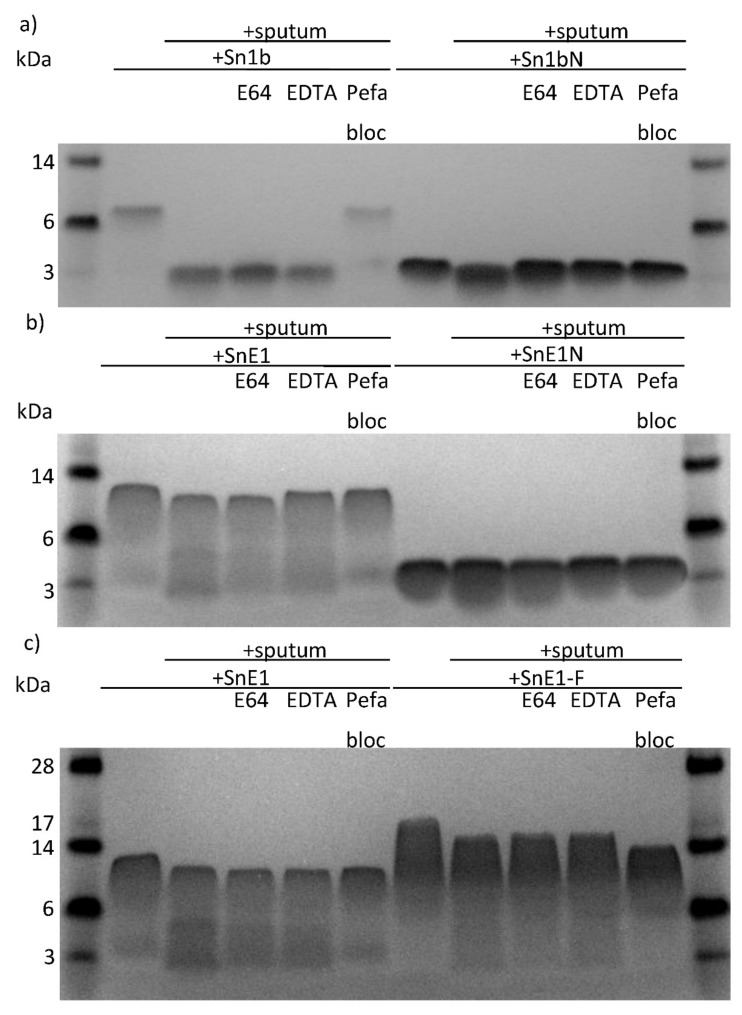

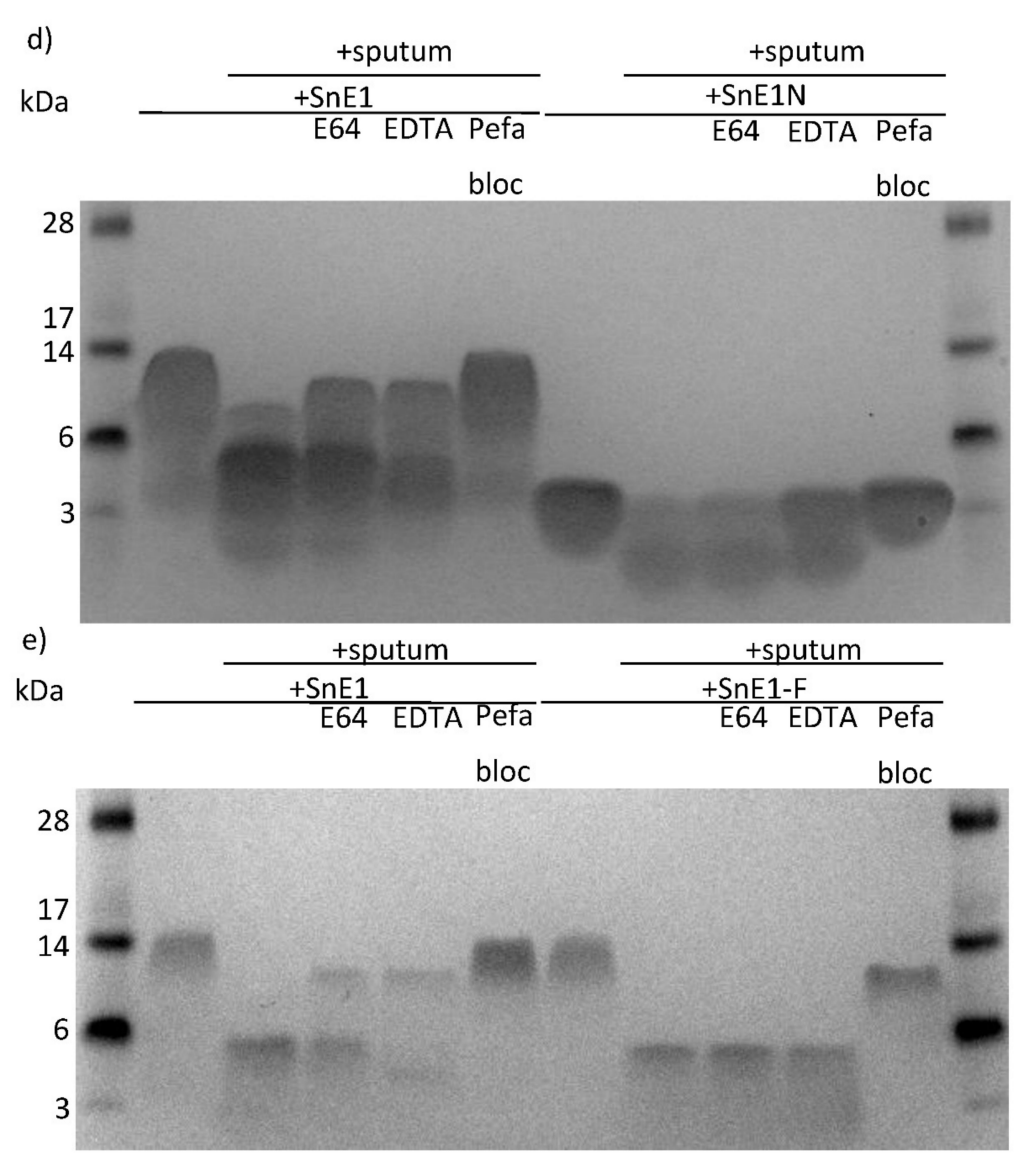
SDS-PAGE gels of next generation peptides following incubation with CF sputum in the presence/absence of protease inhibitors. (**a**) Sn1b, Sn1bN (160 μM), (**b**) SnE1, SnE1N (120 μM), (**c**) SnE1 and SnE1-F (120 μM) were incubated with CF sputum with/without E64, EDTA or pefabloc for four hours. (**d**) SnE1, SnE1N (120 μM), (**e**) SnE1 and SnE1-F (120 μM)*,* were incubated with CF Scheme 64. EDTA or pefabloc for 24 h. Samples were electrophoresed and stained with Coomassie stain for visual analysis.

Peptide derivatives were incubated with sputum in the presence/absence of protease inhibitors and directly compared to respective parent peptides. Sn1bN did not exhibit obvious degradation when incubated with CF sputum after 4 h; whereas parent Sn1b was degraded in the presence of CF sputum, which was inhibited when cysteine, MMP or serine protease inhibitors E64, EDTA or pefabloc, respectively, were present in incubations (Figure 7a). Following 4-hour incubation with sputum with/without inhibitors, some degradation of SnE1-F was observed. This degradation was prevented in the presence of pefabloc (Figure 7b,c). SnE1N and SnE1-F exhibited degradation when incubated with CF sputum for 24 h (Figure 7d,e). Degradation of SnE1 was prevented by pefabloc although E64 and EDTA were also capable of partially inhibiting the degradation of SnE1. EDTA and pefabloc prevented degradation of SnE1N by CF sputum (Figure 7d). Similar to parent SnE1, incubation with pefabloc prevented the degradation of SnE1-F (Figure 7e). Unlike parent SnE1, E64 and EDTA did not prevent SnE1-F degradation.

### 3.8. Peptide Derivatives and NE

**Figure 8:**
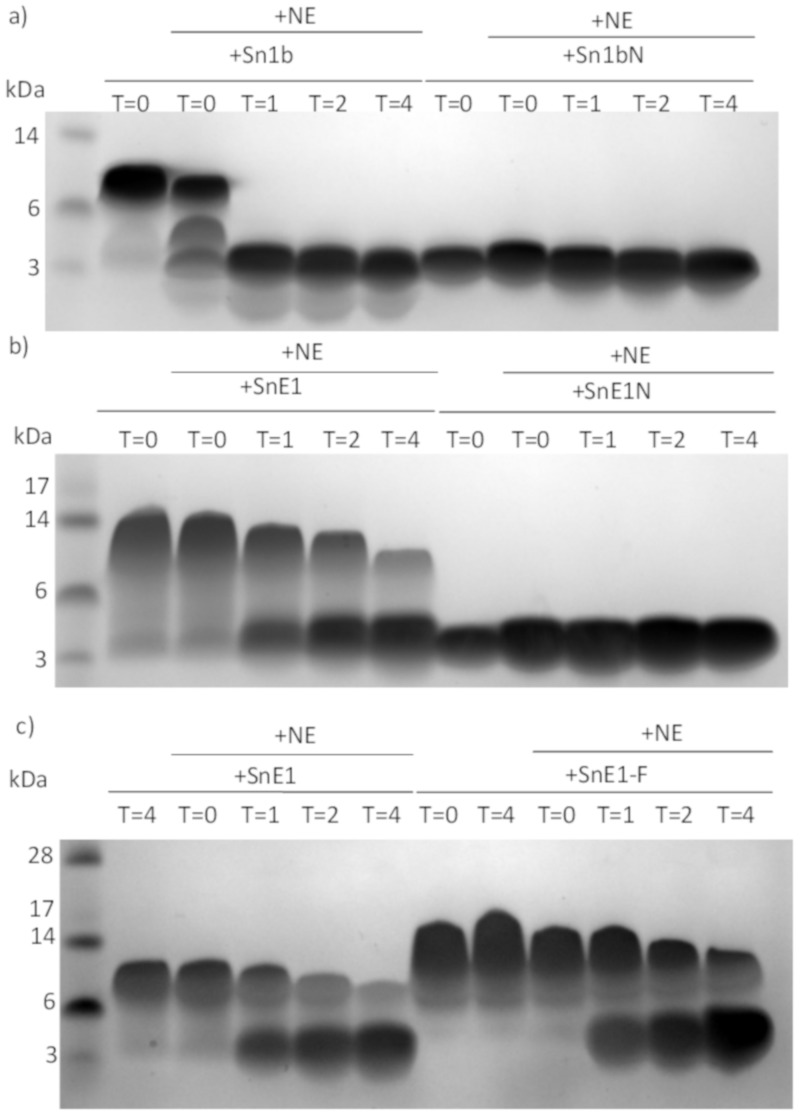
SDS-PAGE gels of next generation derivative peptides following incubation with NE. (**a**) Sn1b, Sn1bN (160 μM), (**b**) SnE1, SnE1N (120 μM) (**c**) SnE1 and SnE1-F (120 μM) were incubated for 0–4 h with 500 nM of neutrophil elastase. Samples were collected at each time-point, electrophoresed and stained with Coomassie stain for visual analysis.

Incubation of Sn1bN with NE did not result in obvious cleavage, whereas parent Sn1b was rapidly cleaved, suggesting Sn1bN was less susceptible to cleavage by NE than parent Sn1b (Figure 8a). SnE1N appeared to remain intact following incubation with NE, unlike parent SnE1, suggesting it is more stable in the presence of NE than the parent (Figure 8b). Similar to parent SnE1, SnE1-F exhibited gradual transition from one band of larger molecular weight to one or more bands of smaller molecular weight (Figure 8c); this observation is to be expected as SnE1-F contains a known NE cleavage site (Table 3).

### 3.9. Effect of NE Incubation on Peptide Derivative Antimicrobial Activity

Sn1bN, SnE1N and SnE1-F were tested using RDAs to determine antimicrobial activity and found to possess potent antimicrobial activity when tested against *P. aeruginosa* 27853 (Table 4). Upon confirmation that the peptide derivatives were susceptible to cleavage by NE, we wanted to determine if cleavage affected the antimicrobial activity of the peptides. Sn1bN, SnE1N and SnE1-F were incubated in the presence/absence of NE for four hours immediately prior to antimicrobial testing using an RDA. 

When tested against *P. aeruginosa* 27853, NE incubation did not result in a statistically significant effect (Table 5); however, Sn1b demonstrated an increase in mean MIC value following NE incubation, suggesting reduced, though still potent, antimicrobial activity. Conversely, Sn1bN exhibited a slight reduction in mean MIC value, suggesting improved antimicrobial activity (Table 5). SnE1 and derivatives SnE1N and SnE1-F exhibited an increase in mean MIC value following NE incubation, again suggesting reduction in antimicrobial activity against this strain. With the exception of Sn1bN exhibiting a slight reduction in mean MIC value, incubation of the peptides with NE led to increased mean MIC values (Table 5); however, observations were not statistically significant. 

### 3.10. Effect of NE Incubation on Derivative Peptide Anti-Inflammatory Activity

**Figure 9:**
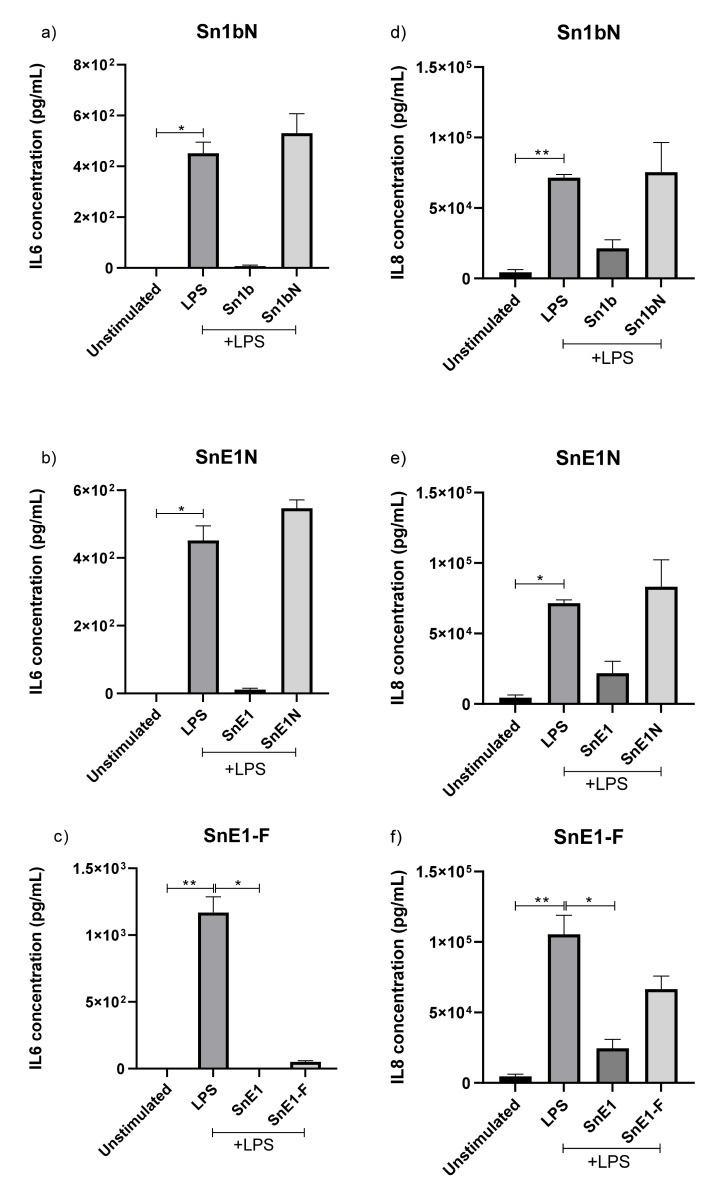
IL-6 and IL-8 concentrations in the supernatant of THP-1 monocyte-derived macrophages following incubation with peptides and LPS. THP-1 monocyte-derived macrophages were incubated with (**a**) Sn1b (1.6 μM), Sn1bN (1.6 μM), (**b**) SnE1 (1.2 μM), SnE1N (1.2 μM), (**c**) SnE1 (1.2 μM) and SnE1-F (1.2 μM) and/or 100 ng/mL of LPS for 16 h. (**a**–**c**) IL-6 and (**d**–**f**) IL-8 levels in cell supernatants were then measured via ELISA. Kruskal–Wallis with Dunn’s Multiple Comparison * *p* < 0.05, ** *p* < 0.01, *n* = 3.

Sn1bN, SnE1N and SnE1-F were tested in an in vitro LPS-induced inflammation model using THP1 monocyte-derived macrophages to compare the impact of truncation on anti-inflammatory activity. Parent peptides Sn1b and SnE1 generated significant or trends toward reduction in LPS-induced IL-6 and IL-8 levels (Figure 9). Derivatives Sn1bN and SnE1N did not alter IL-6 or IL-8 levels, whereas SnE1-F resulted in trends towards reduction in LPS-induced IL-6 and IL-8 (Figure 9). Next, we wanted to determine if incubation with NE affected the anti-inflammatory activity of the peptides. The peptides were incubated with NE for 4 h immediately prior to incubation with LPS-stimulated THP-1 monocyte-derived macrophages. IL-6 and IL-8 in the cell supernatants were measured via ELISA.

**Figure 10:**
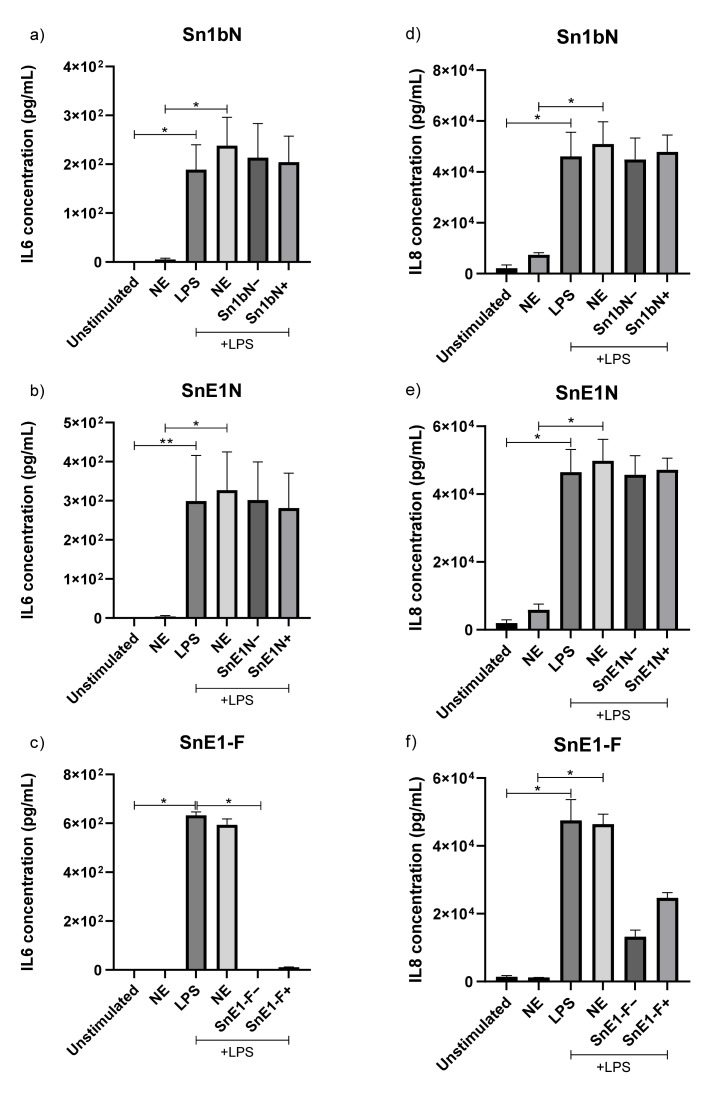
IL-6 and IL-8 concentrations in the supernatants of THP-1 monocyte-derived macrophages following incubation with NE-incubated next generation peptides and/or LPS. THP-1 monocyte-derived macrophages were incubated with/without LPS and/or NE-incubated (**a**,**d**) Sn1bN (1.6 μM), (**b**,**e**) SnE1N (1.2 μM) or (**c**,**f**) SnE1-F (1.2 μM) for 16 h. (**a**–**c**) IL-6 and IL-8 (**d**–**f**) levels in cell supernatants were measured via ELISA. +/− = incubation of peptide in the presence/absence of NE prior to experiment. Kruskal–Wallis with Dunn’s Multiple Comparison, * *p* < 0.05, ** *p* < 0.01. *n* = 2–4.

Sn1bN and SnE1N had no effect on LPS-induced IL-6 or IL-8 levels, regardless of NE incubation status (Figure 10a,b,d,e). Only SnE1-F incubated in the absence of NE prior to cell stimulation significantly reduced LPS-induced IL-6 levels (Figure 10c). 

## 4. Discussion

Individuals with chronic inflammatory lung diseases are more susceptible to infection, with up to 95% of CF patients ultimately succumbing to chronic lung infection [2]. Snake-derived AMPs possess therapeutic potential due to their antimicrobial and anti-inflammatory properties [14,16,19]. When assessing the therapeutic potential of a peptide in the context of chronic inflammatory lung disease, it is important to consider the local microenvironment to which they will be exposed. The lungs of individuals with inflammatory lung diseases, such as CF or COPD, often exhibit impaired innate immune defence, featuring neutrophilic inflammation, mucus hypersecretion, impaired bacterial clearance and protease release [24]. In this study, we assessed the effect of sputum proteases on snake-derived AMPs. 

Sputum is a heterogeneous mixture of cells and mucus expelled from the lower airways by coughing [25]. Neutrophilic infiltration is characteristic of CF and neutrophil-derived proteases, including NE, proteinase 3 and cathepsin G, have been detected in the sputum and/or BAL fluid of CF patients [26,27]. Many AMPs, including LL37, human β-defensins-2 and -3, SLPI and elafin, are susceptible to degradation by proteases including NE and cathepsins [28,29,30,31,32]. The presence of proteases in the lung microenvironment poses an issue for the stability and longevity of endogenous and exogenous AMPs alike. 

In this study, the snake-derived AMPs Sn1, Sn1a, Sn1b, SnE1 and SnV1 were suspected to be susceptible to cleavage by lung relevant proteases, most notably, NE. This suspicion was confirmed by SDS-PAGE of peptide-sputum incubation samples. Further incubation with CF sputum in the presence/absence of serine, cysteine and MMP protease inhibitors highlighted that serine proteases were the major hydrolytic enzymes responsible for the observed degradation (Figure 2).

Compared to the other peptides, SnE1 exhibited little degradation within 4 h, suggesting superior stability (Figure 1c). However, degradation was evident after 18 h of incubation with CF sputum, and complete degradation was observed after 24 h (Figure 1e). These findings suggest SnE1 is susceptible to degradation by serine proteases, but is more stable than the other peptides of interest. 

Rapid cleavage was observed when peptides were incubated with NE. Similarly, other peptides including LL37, elafin and SLPI have been shown to be susceptible to cleavage by human CF lung proteases such as NE, resulting in inactivation [29,30,32]. NE incubation of Sn1b, SnE1 and SnV1 slightly reduced mean MIC values but these still demonstrated potent antimicrobial activities (Table 2). On the other hand, Sn1, Sn1a, Sn1b and SnV1 did not alter LPS-induced IL-6 or IL-8 release following NE incubation, suggesting peptide cleavage causes loss of anti-inflammatory activity (Figure 4). By contrast, incubation of LPS-stimulated THP-1 cells with NE-treated SnE1 resulted in a significant reduction in IL-6 levels and a trend toward reduction in IL-8 levels, similar to that observed with the untreated peptide (Figure 4). These findings suggest that while NE cleavage reduced the antimicrobial ability of Sn1 and Sn1a and abolished anti-inflammatory activity, Sn1b and SnV1 retained antimicrobial activity but lost anti-inflammatory activity; whereas, SnE1 cleavage products retained both antimicrobial and anti-inflammatory functions. 

Mass spectrometric analysis confirmed that Sn1b, SnE1 and SnV1 were cleaved by NE at valine or isoleucine residues (Figure 5), which is in agreement with a study conducted by O’Donoghue et al. (2013) [33]. Identifying amino acid sequences possessing antimicrobial and/or anti-inflammatory activity in microenvironments containing NE was desirable as shorter peptides are subject to lower production costs and less likely to be immunogenic [34]. 

Surprisingly, peptide derivatives demonstrated similar antimicrobial activity to respective parents (Table 5). Sn1bN and SnE1N are 14 amino acid long peptides with +7 net charge; they are derived from the predicted α-helical N-termini of their respective parent peptides (Table 5). Another short snake peptide derivative, OH-CATH(3–17) is almost identical to SnE1N but is one amino acid residue longer, beginning at the third amino acid residue of SnE1 instead of the first and has a +8 net charge [35]; it is also very similar to Sn1bN. OH-CATH(3–17) has been shown to be antimicrobial against a range of bacterial strains, including *P. aeruginosa* 27853 (MIC 4.15 μM) [35]. In this study, SnE1N and Sn1bN were more effective against this strain, with MIC values of 0.489 μM and 0.700 μM, respectively (Table 5). Therefore, these combined findings support the general consensus that cationic, amphipathic α-helical structures are important for antimicrobial activity. However, crotalacidin(1–14) a 14 amino acid derivative of parent Crotalicidin (known here as SnV1) with a +8 net charge, contradicts this view [16]. Despite possessing a highly similar amino acid sequence and structure to Sn1bN and SnE1N, Ctn(1–14) did not exhibit antimicrobial activity against a variety of bacterial strains, including *P. aeruginosa* 27853 [16]. However, these contrasting data may be due to the use of a microdilution broth antimicrobial assay. Zhang and colleagues (2010) reported that AMPs containing less than 24 amino acid residues may be readily inactivated or bound to anionic components of nutrient broth, leading to the inaccurate conclusion that peptides lacked antimicrobial capacity [35]. Utilisation of an RDA may yield different results.

It has been frequently described that a minimum length is required for antimicrobial activity of AMPs [36,37,38,39]. A minimum length of 12 amino acid residues to accomplish three α-helix turns is a common requirement among various AMPs [36,38,39]. The presence of an α-helical structure is considered an important aspect for AMP activity. When Wei et al. (2015) generated a scrambled Sn1 peptide (sHc-CATH) with an identical amino acid composition and net charge to the parent peptide, without an α-helical structure, sHc-CATH was shown to possess no antimicrobial or LPS neutralising activity, suggesting that the presence of α-helices was essential for function [14]. Blondelle and Houghten (1992) reported that an amphipathic peptide of 12–18 residues was necessary for antibacterial activity, with 14 amino acids displaying optimal activity [36]. More relevantly, a study of Hc-CATH (known here as Sn1) truncated peptides revealed that antibacterial activity decreased below 16 amino acids in length, with almost no activity evident at 13 amino acid residues long [40]. Similarly, in this study peptides composed of 14 amino acid residues were capable of potent antimicrobial activity. While incubation with NE did not significantly alter the antimicrobial activity of the truncated peptide derivatives compared to incubated peptide controls, a slight decrease in antimicrobial activity was observed following four-hour incubation of peptide derivatives at 37 °C prior to RDAs compared to freshly prepared peptide, whereas parental peptide MIC values were similar to freshly prepared peptide.

Highly cationic peptides may be capable of binding anionic LPS molecules but may be insufficient for LPS aggregate dissociation and/or inhibition of an LPS-induced inflammatory response [41,42,43]. The ability of peptides to neutralise LPS is affected by a combination of factors including net charge, hydrophobicity, amphipathicity, the presence of aromatic residues, and peptide structure (Schmidtchen et al., 2014). Peptide truncation can result in an imbalance between these factors, leading to altered antimicrobial activity and LPS-binding ability [39,44,45,46].

The trends observed when NE-incubated parent peptides were tested in an in vitro LPS-induced inflammation model allowed prediction of truncated peptide derivative activity. Given the lack of anti-inflammatory activity of NE-incubated Sn1b, the lack of anti-inflammatory effect of Sn1bN was to be expected whereas, SnE1N and SnE1-F activities were more difficult to anticipate due to the increased number of cleavage products (Table 3). Only the derivative SnE1-F demonstrated anti-inflammatory activity in the in vitro LPS-induced inflammation model (Figure 9). Many AMPs, including Hc-CATH (Sn1), are capable of LPS binding and neutralisation and it is possible that the peptides in this study may share this property [14,43,47,48]. LPS binding alone does not guarantee inhibition of the LPS-induced inflammatory response [43]. This is exemplified by the LL37 derivative KR-12 that was shown to bind directly to LPS in a similar manner to the parent peptide, but did not share the ability to inhibit LPS-induced inflammation in vitro [41,42].

It is likely that a combination of differing physiochemical characteristics between derivative and parent peptides is responsible for the observed differences in activities (Schmidtchen et al., 2014). Sn1bN is predicted to be more hydrophobic and amphipathic than Sn1b, and with a lesser net charge of +7 compared to +14 (Table 1). While increased hydrophobicity and amphipathicity are thought to enhance LPS-binding, reduction in cationicity is expected to reduce the interaction [14,44,45]. For example, Wei and colleagues (2015) demonstrated that gradual reduction in antimicrobial and anti-inflammatory activity of Hc-CATH corresponded with gradual reduction in net cationic charge [14] The presence of fewer aromatic residues is also associated with less effective LPS-binding [39,49,50]. It is possible that the enhanced interaction that may be expected to arise from increased hydrophobicity and amphipathicity may be insufficient to overcome the decreased interaction arising from loss of stabilising aromatic residues, reduction of cationicity, and the shortened α-helical region arising from the reduction of overall peptide size. On the other hand, SnE1N is predicted to be less hydrophobic and cationic, and to possess fewer aromatic residues, but is expected to be more amphipathic than parent SnE1. SnE1-F is predicted to be even less hydrophobic, more amphipathic and to possess one less aromatic residue than SnE1, with an identical net charge and almost identical predicted alpha helical content. For SnE1, reduction of cationicity, length and number of phenylalanine residues may converge to reduce LPS neutralisation. Overall, it is reasonable to speculate that reduction of cationicity, α-helical content and/or the number of aromatic residues of Sn1b and SnE1 negatively impact the capacity for LPS neutralisation; whereas, overall hydrophobicity does not appear to be the most dominant factor.

## 5. Conclusions

In summary, we have shown that varied snake-derived AMPs are susceptible to proteolytic degradation of lung proteases, particularly NE. However, this degradation may not necessarily disrupt their anti-microbial or immune modulatory functions. We have exploited this latter fact to design new, shorter AMPs that are more resistant to degradation which may have improved therapeutic effects while at the same time been more amenable to synthesis, less immunogenic and more affordable.

## Figures and Tables

**Table 1:** Predicted physiochemical properties of all peptides utilised in this study. Physiochemical properties of the peptides were predicted using ExPASy Bioinformatics Resource Portal Protparam software available at https://web.expasy.org/protparam/ (accessed on 26 July 2021).

Peptide	Amino Acid Sequence	Molecular Weight (Da)	Net Charge at pH 7	Hydrophobic Moment	GRAVY
Sn1	KFFKRLLKSVRRAVKKFRKKPRLIGLSTLL	3628.59	+12	0.425	−0.273
Sn1a	KFFKRLLKSVRRAVKKFRKK	2564.25	+11	0.723	−0.995
Sn1b	KRFKKFFKRLLKSVRRAVKKFRKK	3123.97	+14	0.726	−1.225
Sn1bN	KRFKKFFKRLLKSV	1825.32	+7	0.831	−0.650
SnE1	KRFKKFFKKLKNSVKKRAKKFFKKPRVIGVSIPF	4155.23	+15	0.457	−0.735
SnE1N	KRFKKFFKKLKNSV	1798.25	+7	0.768	−1.129
SnE1-F	KRFKKFFKKLKNSVKKRAKKFFKKPRVI	3554.51	+15	0.599	−1.204
SnV1	KRFKKFFKKVKKSVKKRLKKIFKKPMVIGVTIPF	4152.37	+15	0.440	−0.435

**Table 2:** Mean (±SEM) MIC values against *P. aeruginosa* 27853. Peptides were incubated with/without NE for four hours prior to RDA. Mann–Whitney, peptide alone vs. peptide + NE. ^a^ *p* = 0.0317.

Peptide	MIC (μM ± SEM) against *P. aeruginosa* 27853		
	Peptide Alone	Peptide + NE	Fold Change in MIC
Sn1 (*n* = 3)	4.41 ± 2.16	20.0 ± 7.33	4.54
Sn1a (*n* = 3)	0.660 ± 0.423	45.6 ± 3.10	69.1
Sn1b (*n* = 4)	3.59 ± 2.13	7.31 ± 3.36	2.04
SnE1 (*n* = 4)	3.25 ± 1.96	5.07 ± 3.85	1.56
SnV1 (*n* = 5)	0.877 ± 0.434	5.42 ± 1.59 ^a^	6.18

**Table 3:** Peptide neutrophil elastase cleavage sites, indicated by amino acid in bold and underline, as determined by mass spectrometry profiles of NE-incubated peptides. Peptides are cleaved after each cleavage site indicated.

Peptide	Amino Acid Sequence
Sn1b	KRFKKFFKRL**L**│KS**V**│RRAVKKFRKK
SnE1	KRFKKFFKKLKNS**V**│KKRAKKFFKKPRV**I**│GVSIPF
SnV1	KRFKKFFKK**V**│KKSVKKRLKK**I**│FKKPMVIGVTIPF

**Table 4:** MIC values of parent and next generation peptides. MIC values of next generation and parent peptides when tested against *P. aeruginosa* 27853 using RDAs.

Peptide	Mean MIC Value (μM) ± SEM against *P. aeruginosa* 27853
Sn1b (*n* = 5)	2.20 ± 0.673
Sn1bN (*n* = 5)	1.39 ± 0.725
SnE1 (*n* = 9)	1.92 ± 1.13
SnE1N (*n* = 6)	0.489 ± 0.160
SnE1-F (*n* = 3)	0.410 ± 0.310

**Table 5:** MIC values of NE-incubated next generation peptides and parents against *P. aeruginosa* 27853 following NE incubation. MIC values of NE-incubated peptides against *P. aeruginosa* 27853 as determined by RDAs. Peptides were incubated with/without 500 nM NE for four hours prior to RDAs.

Peptide	MIC (μM ± SEM) against *P. aeruginosa* 27853		
	Peptide Alone	Peptide + NE	Fold Change in MIC
Sn1b (*n* = 4)	3.59 ± 2.13	7.31 ± 3.36	2.04
Sn1bN (*n* = 2)	5.32 ± 4.16	5.09 ± 4.31	0.957
SnE1 (*n* = 4)	3.25 ± 1.96	5.07 ± 3.85	1.56
SnE1N (*n* = 3)	4.28 ± 2.03	6.54 ± 3.87	1.53
SnE1-F (*n* = 2)	2.04 ± 0.315	4.84 ± 1.31	2.37

## Data Availability

The data presented in this study are available on request from the corresponding author. The data are not publicly available due to space restrictions.
